# High-SETD8 inactivates p53 in neuroblastoma

**DOI:** 10.18632/oncoscience.344

**Published:** 2017-04-14

**Authors:** Veronica Veschi, Carol J. Thiele

**Affiliations:** Cell and Molecular Biology Section, Pediatric Oncology Branch, Center for Cancer Research, National Cancer Institute, Bethesda, MD, USA

**Keywords:** neuroblastoma, SETD8, p53, epigenetics

Epigenetics and differentiation are intimately related in Neuroblastoma (NB), the most common extracranial solid tumor of childhood. NB originates from neural crest and primary tumors have a sympathoadrenal progenitor phenotype. High-risk NB is considered a failure of sympathoadrenal terminal differentiation. Despite intense multimodal treatment, high-risk NB is also one of the most aggressive pediatric tumors accounting for 15% of all pediatric oncology deaths with less than 50% of patients experiencing long-term survival. Given the findings that epigenetic drivers contribute to NB tumorigenesis and the scarcity of druggable somatic mutations, we performed a chromatin-focused siRNA screen in order to study in an unbiased way the epigenetic landscape of NB tumors. By using a high-content Opera imaging platform, of the 400 genes analyzed we uncovered 53 epigenetic regulators critical for survival of high-risk NBs, with 16 crucial for NB differentiation. We further screened 21 epigenetic compounds in 8 NB cell lines to prioritize siRNA hits that are in the drug developmental pipeline.

Integration of high-throughput genetic and chemical-based screen data revealed SETD8, as an important and druggable target in NB [1]. SETD8 is the sole methyltransferase that catalyzes monomethylation of lysine 20 on histone H4 (H4K20me1), regulating DNA replication, chromosome condensation and gene expression. The chemical screen identified the SETD8 inhibitor UNC0379 [2] as one of the most active and selective compounds in inhibiting NB cell growth. In primary neuroblastoma tumors, SETD8 RNA levels were significantly associated with poor prognosis in high-risk NBs, specifically in MYCN-wild-type stage 4 patients. SETD8 also monomethylates non-histone proteins, such as the tumor suppressor p53. The SETD8-mediated monomethylation on lysine 382 of p53 (p53K382me1) attenuates the p53 pro-apoptotic and growth arrest functions [3]. RNA-seq and functional studies indicated that genetic or pharmacological inhibition of SETD8 activity led to activation of the p53 canonical pathway in NB by decreasing p53K382me1 levels [1]. Specifically, SETD8 inhibition resulted in decreased p53K382me1 and H4K20me1, accompanied by increased p53 total protein and p21, reduced cell growth and increased cell death. Genetic rescue experiments confirmed that SETD8- induced cell death is p53 dependent and p53K382 is important for this activity. P53 is rarely mutated in primary NB (<2%). Several mechanisms have been identified in MYCN amplified NB tumors that functionally “inactivate” p53 and these primarily result from the impact of MYCN amplification induced increases in MDM2 and HMGA1 [4–6]. Our identification of SETD8-mediated decreases in p53 activity is a novel mechanism in NB and aside from mutations or epigenetic repression of p14ARF [7] represents the first that is relevant to the MYCN-wild-type NBs. These tumors account for some 60-70% of high-risk NB tumors.

Our *in vivo* xenograft NB models, showed that genetic or pharmacologic (UNC0379) inhibition of SETD8 impairs *in vivo* outgrowth of NB xenograft tumors and confers a significant survival advantage. This study identifies that SETD8 is a novel therapeutic target and its inhibition may be especially relevant for the subset of high-risk NB tumors with wild-type MYCN. This is the first *in vivo* preclinical study showing that targeting SETD8 inhibits tumor growth.

SETD8 has been found overexpressed or upregulated in many different types of tumors such as bladder cancer, non–small cell lung carcinoma, small cell lung carcinoma, chronic myelogenous leukemia, hepatocellular carcinoma and pancreatic cancer [8]. This novel mechanism of p53 inactivation mediated by SETD8 may be relevant in other pediatric and adult tumors with p53 wild-type and SETD8 overexpression. Small molecule inhibitors of SETD8 may lead to p53 reactivation in such settings and may offer a novel therapeutic approach when combined with conventional cytotoxic agents.
